# Promotion and preservation of mobility and autonomy in old age through smart rollators—a qualitative study

**DOI:** 10.3389/fdgth.2026.1783585

**Published:** 2026-04-20

**Authors:** Patrick Schwaiger, Julia Müller, Martin Obert, Stefan Twieg, Andreas Butkov, Ulrike Steinmann, Denny Paulicke, Patrick Jahn

**Affiliations:** 1 Department of Internal Medicine, Faculty of Medicine, University Medicine Halle (Saale), Research Group for Health Services Research, Nursing in Hospitals, Martin Luther University Halle-Wittenberg, Halle (Saale), Germany; 2 Department of Electrical Engineering, Mechanical Engineering and Industrial Engineering, Anhalt University of Applied Sciences, Köthen, Germany; 3 Faculty of Electrical and Information Engineering, Otto von Guericke University Magdeburg, Institute of Automation Engineering, Magdeburg, Germany

**Keywords:** assistive technologies, design-based research, nursing, participatory development, smart rollators

## Abstract

**Background:**

Diseases and health limitations associated with ageing often result in loss of mobility and reduced social participation. The ongoing demographic shift towards an increasingly ageing population, combined with a declining number of healthcare professionals, highlights the need to integrate digital assistive solutions to reduce workload and healthcare costs. Smart rollators (SRs) equipped with sensor-based assistance systems (SAS) are considered a promising innovation for enhancing safety, mobility, and independence in older adults.

**Objective:**

The aim of this study was to explore the needs, experiences, and perspectives of rollator users (RU) and healthcare professionals (HP) in order to identify and evaluate user-centred requirements for the iterative development of a smart rollator.

**Methods:**

As part of a broader research project, a design-based research (DBR) approach was applied. Five focus groups with a total of 30 participants (15 RU, 15 HP) were conducted using semi-structured interviews, which were analysed using qualitative content analysis. Data collection occurred in two phases: first, to explore user requirements, and subsequently, to evaluate an initial SR prototype. The analysis followed a content-structuring approach, conducted independently by two researchers.

**Results:**

Three main categories emerged from the focus groups: use of the rollator in daily life, sensor-based assistance systems, and the application of digital assistive technologies. Participants generally assessed the integration of digital and sensor-based functions positively, provided that these increased perceived safety and remained easy to use. Desired features included navigation, environmental and fall detection, emergency call functionality, lighting, and haptic feedback. Barriers were primarily related to technological scepticism, limited digital literacy, and potential cognitive overload. Facilitating factors included training, simple user interfaces, and modular system structures.

**Conclusion:**

The findings indicate that participatory development processes are essential for improving the acceptance of smart rollators. Early involvement of users and nursing professionals ensures that technological innovations are designed to be practical, safe, and needs-oriented. The design-based research approach represents a suitable framework for the iterative development and evaluation of assistive technologies in healthcare settings.

## Introduction

The projected increase in the global population, particularly within the 60+ age group — expected to reach nearly 2.1 billion by 2050 due to demographic change — presents a wide range of challenges for healthcare systems (1–3). As life expectancy increases, the likelihood of age-related diseases (1, 4, 5) and the demand for medical and nursing care (6) rise accordingly, resulting in higher economic costs for healthcare institutions and insurance providers (7–10). The diverse physiological changes associated with ageing affect sensory, musculoskeletal, neurocognitive, and cardiovascular functions (4, 5). In particular, mobility impairments and reduced physical activity — which typically lead to further declines in functional capacity (4) — often exacerbate underlying conditions, diminish quality of life, and limit the ability to perform basic daily activities (1, 11). To varying degrees, mobility and balance in older adults are negatively affected by these age-related changes, resulting in altered gait patterns and reduced gait quality (12). Consequently, assistive devices such as walking sticks or rollators are commonly used to compensate for individual limitations (13).

In recent years, with the increasing digitalisation of nearly all areas of life, there has been extensive research in the field of assistive robotics. Within this context, a variety of approaches have been developed to explore how smart rollators (SRs) equipped with sensor-based assistance systems (SAS) can support older adults with mobility or cognitive impairments in daily life (14–16) Conventional rollators without intelligent assistance functions often fail to meet users' individual needs (17). Previous research has primarily focused on analysing gait patterns during daily activities or fall-free walking in rehabilitation settings (18–22). Additional functions such as sensor-based environmental perception (23–27) and navigation support (15, 25, 26, 28) are intended to enhance safety during everyday mobility and prevent collisions with obstacles. Some studies have also examined the extent to which SAS can contribute to fall prevention and detection (29–31). Most previous research has concentrated on how SRs can serve as physical aids for users in daily life or rehabilitation. However, the transferability of findings remains limited, as many studies were conducted either with healthy participants (29, 32–34), with small sample sizes (16, 22, 35), or as technical development studies without patient involvement (17, 28, 30, 36). A further challenge in SAS development lies in the fact that end users are often not involved in the design process but only included at the evaluation stage. Yet, users' individual requirements and needs are complex and must form the basis for SR development to ensure user-friendly operation, acceptance, and ultimately the promotion of mobility and independence among older adults (3, 31, 32, 37–42). The study by Aruona et al. (42) follows precisely this approach, investigating the perspectives of older adults and healthcare professionals regarding existing rollators. More than 50% of respondents identified a need for innovative systems with improved navigation and ergonomic design in commercially available rollators. This demand appears justified, as only 55% of older participants reported feeling safe when using their rollator, and 59% considered it suitable for outdoor use. Another study by Orenius et al. (39) demonstrated that intelligent rollator functions had no significant effect on participants' motivation to use the device. However, those with prior rollator experience showed a greater willingness to engage with SR feedback and reported increased motivation for physical activity compared to first-time users (39). Recent research on SRs (23, 34) has adopted participatory, co-creative development approaches that incorporate qualitative insights from healthcare experts during the design process. In this context, the user is no longer seen merely as a passive recipient of physical support through SAS, but rather as an active participant interacting dynamically with the SR (26, 34). The use of multimodal assistance systems addresses not only mobility support but also, for the first time, the social well-being of users (34). Existing SRs that have reached the commercial market, such as the *e-Rollator* developed from the concept of Bieber et al. (24), remain highly costly and thus accessible only to a minority of individuals. In addition to financial and regulatory constraints (32, 43) in SR development, it is essential to strengthen the knowledge and competencies of users and professionals regarding assistive technologies (3) to enhance acceptance of such devices (37). When tailored to users' needs and environmental contexts, and when individual impairments are taken into account, assistive technologies have the potential to maintain autonomy and functional ability in older adults.

Many age-related changes can be positively influenced through targeted lifestyle adjustments, which may be facilitated by the use of assistive technologies (3). A smart rollator designed to promote and maintain mobility and physical activity holds great potential to enhance both the cognitive and physical health of older adults (11, 44, 45), while simultaneously reducing the workload of healthcare professionals (3, 16, 24, 37). Accordingly, the present study aimed to identify and integrate the needs, experiences, and perspectives of rollator users (RU) and healthcare professionals (HP) into the development of a smart rollator, employing a design-based research (DBR) approach. Semi-structured interviews conducted in focus groups served to capture user perspectives, with a particular focus on the utilisation and application of digital assistive technologies.

## Methods

This study investigates which user-centred needs and requirements digital assistance systems should meet when added to a conventional rollator, and which opportunities and challenges may arise for future care provision. At project outset, a design-based research (DBR) approach was chosen (46). The core rationale of this approach is to systematically end users' practical perspectives into the development process through participatory collaboration between researchers and stakeholders, thereby increasing acceptance of the artefact being developed. The iterative process was implemented using semi-structured, guide-based focus groups (FG) (47). Qualitative data analysis followed the content-analytical approach described by Kuckartz et al. (48).

### Participants

For the purposes of the development process, the focus was primarily on recruiting rollator users (RU) aged over 65 years and healthcare professionals (HP) who are actively and regularly involved in patient care. All participants (PT) were required to be able to communicate and to have sufficient proficiency in German. Potential PT with substantial cognitive impairment (e.g., due to dementia) or those in acute inpatient treatment were not eligible for inclusion (see Table 1). No prior relationship existed between the research team and the PT before the study commenced. Recruitment was carried out by contacting various care facilities in Saxony-Anhalt via email and telephone. Interested individuals received project information materials, a description of the focus group procedure, and an informed consent form by email.

**Table 1:** Inclusion and exclusion criteria for rollator users (RU) and healthcare professionals (HP).

Inclusion criteria	Exclusion criteria
Age ≥ 18 years	Currently undergoing acute hospital treatment
Require a walking aid at the start of the study	Exhibit significant cognitive impairments (e.g., dementia)
Able to communicate and possess sufficient proficiency in written and spoken German (RU & HP)	—
Have provided written informed consent to participate after a detailed explanation of the study (RU & HP)	—
Actively and regularly involved in patient care (HP)	—

### Data collection

Following a systematic literature review (49), semi-structured interview guides were developed through an iterative peer group process within the research team. These guides covered the topics “User behaviour, conditions, and experiences in the use of rollators”, “Requirements, framework conditions, and implementation possibilities of digital assistive technologies”, and “Requirements for digital assistive technology” (50) They served as the basis for the semi-structured interviews with rollator users (RU) and healthcare professionals (HP). Within the research group, an iterative and quality-assured peer review process was conducted in advance to refine the focus group guides. During the first phase (T0), from November 2024 to January 2025, three focus groups were carried out. The insights gained were used to inform the development of individual assistance system components. In a second phase (T1), from June to July 2025, a first prototype was presented and discussed in two additional focus groups. The findings and feedback provided by participants were directly incorporated into the ongoing prototype development.

All five focus groups were conducted in person and audio-recorded. Each session lasted approximately 120 min and was moderated by one researcher, accompanied by another researcher responsible for the technical development of the specific assistance function being discussed. An additional research team member was responsible for taking field notes.

### Data analysis

The qualitative data were fully transcribed based on the audio recordings. Transcription was carried out according to the established rules of Dresing & Pehl (51), ensuring a standardised and transparent written representation of the discussions. The data were then analysed using the content-structuring qualitative content analysis method described by (48). Two researchers conducted the analysis independently to ensure traceability and reliability of the results.

After initial coding, intercoder comparisons were performed to identify discrepancies in category assignment. Divergent interpretations were discussed in structured consensus meetings and resolved through joint re-examination of the respective text passages. If necessary, a third researcher was consulted. Coding decisions and category refinements were documented to ensure transparency and consistency of the final category system. In this multi-stage process, deductive main categories were first defined based on the research questions and subsequently expanded with inductively developed subcategories during analysis. Differences in categorisation were discussed within the research team and resolved by consensusbuilding procedures.

### Prototype

Based on insights from the literature and findings from the semi-structured interviews with rollator users (RU) and healthcare professionals (HP), the following sensor-based assistance systems (SAS) were developed. From a functional perspective, the integration of these SAS modules into a conventional rollator was guided strongly by participants' feedback. The individual user requests recorded from the focus groups were taken into account in that, for example, the different positioning of the individual sensors was discussed with the participants in terms of technical functionality, user-friendliness, and acceptance. For the haptic feedback, various vibration modes and intensities were first demonstrated to the participants using a model in the initial stage, and the texture and design of the future handles were discussed. After six months, the first prototype was then presented together with the user interface as a complete system, in which the individual assistance systems were tested in practice by the participants and discussed again. Involving end users ensured that individual requirements were adequately considered, thereby enhancing the acceptance of the system. The expertise of HP regarding the everyday challenges faced by older adults using rollators—particularly issues of mobility, usability, potential implementation barriers, and expected obstacles in the everyday use of assistive technologies (AT)—was likewise indispensable.

To operationalise the DBR approach, qualitative findings from each focus group phase were systematically translated into design requirements. Identified categories were condensed into structured requirement statements and prioritised based on relevance and technical feasibility. In interdisciplinary meetings, these requirements were directly linked to specific system components (e.g., navigation, haptic feedback, emergency call), and implementation decisions were documented. Prototype adaptations in T1 were explicitly derived from user feedback collected in T0, ensuring iterative refinement grounded in empirical data.

**Figure 1:**
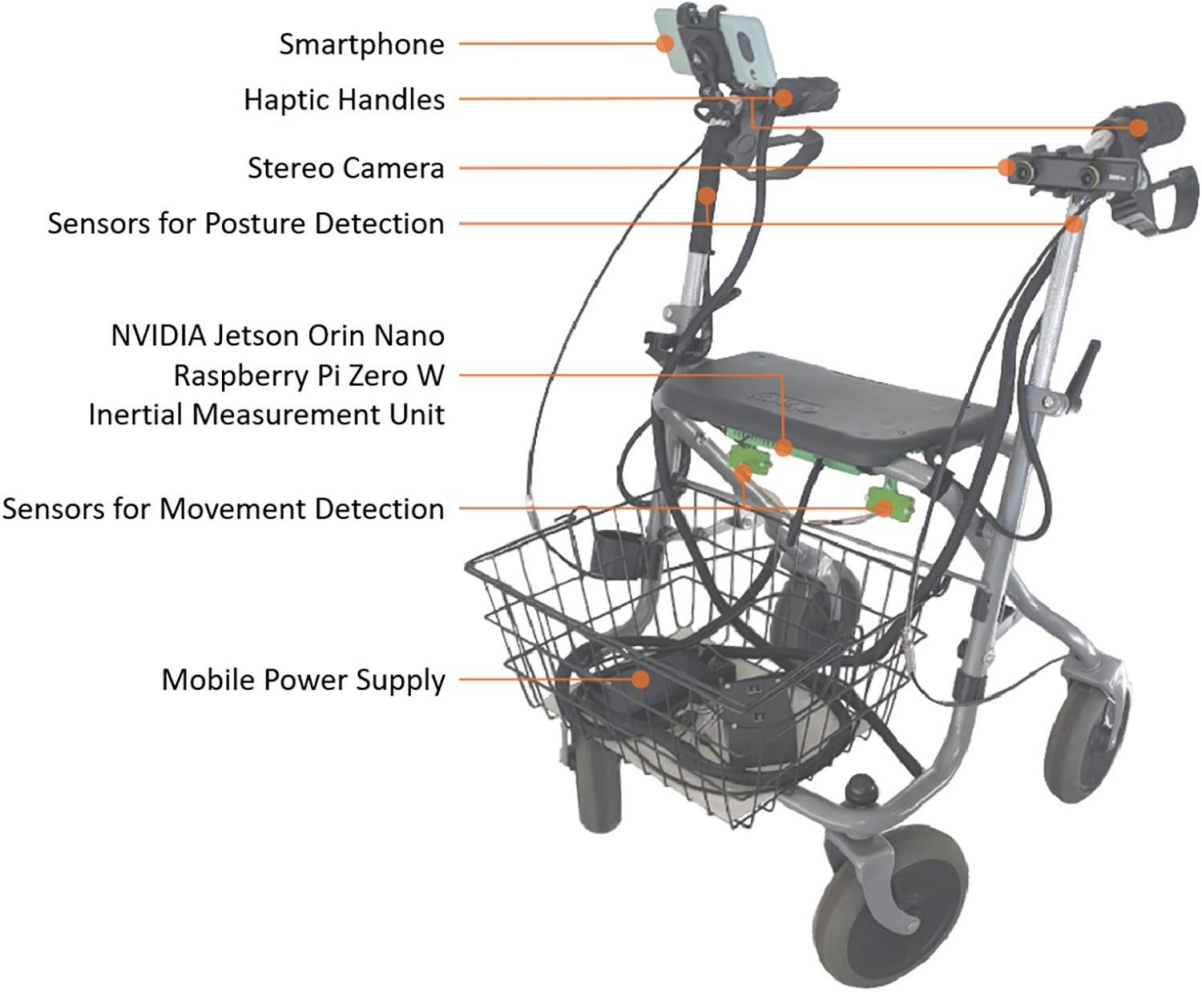
Smart rollator prototype with built-in sensor-based assistance systems.

**Figure 2:**
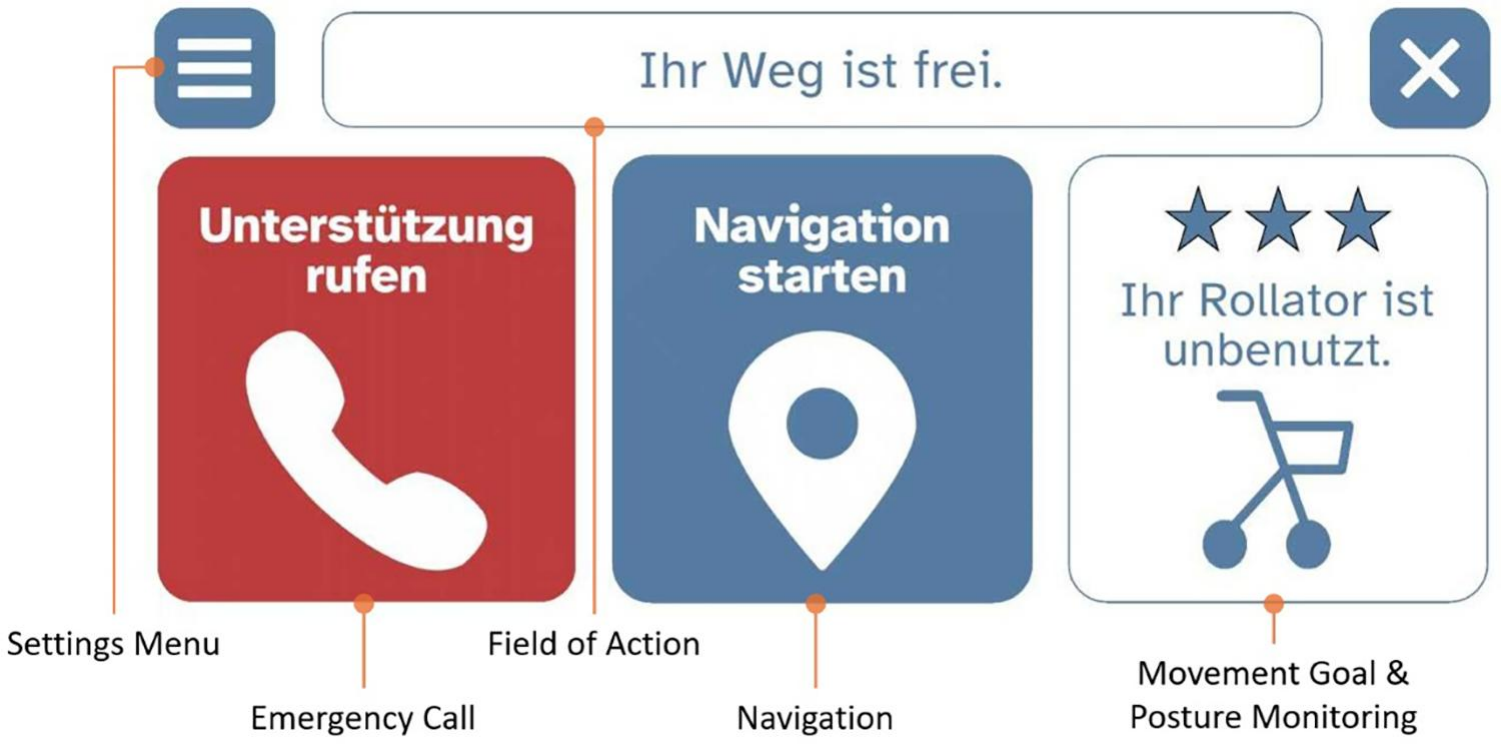
User interface of the smart rollator.

For this study, a conventional rollator was equipped with several modular sensors and feedback components to provide visual and haptic user interface functions, supporting the user in everyday life as part of a smart rollator concept. The individual SAS components are shown in Figure 1 and the developed user interface (UI) is shown in Figure 2.

The prototype combines a high-performance edge AI platform with stereoscopic imaging, supplementary posture detection sensors, and an MQTT-based data bus. The computational platform consists of an NVIDIA Jetson Orin Nano mounted beneath the seat, housed together with a Raspberry Pi Zero W in a protective 3D-printed device carrier. Environmental perception is achieved through a Stereolabs ZED Mini stereoscopic camera attached via a custom-designed mount at the curved section of the handlebar. Additional posture and motion detection sensors are installed at the grips. Sensor data are transmitted via MQTT, with the NVIDIA Jetson Orin Nano serving as the MQTT broker and performing YOLOv11-based inference for object detection, segmentation, and tracking.

To provide haptic feedback, a modified version of the original handle was developed. The handle was 3D-printed from thermoplastic polyurethane (TPU) and features six recesses (three on the upper and three on the lower side), each housing vibration motors acting as actuators for haptic feedback. To achieve mechanical decoupling from the handle, spring-based damping elements were installed beneath each actuator, ensuring precise localisation of feedback. The developed user interface (UI) includes core functions such as navigation, emergency call, and system status, as well as an output line and settings menu. In the navigation section, predefined destinations (e.g., care home, general practitioner, pharmacy) can be selected by the user during movement of the SR, or custom destinations can be entered manually. The same principle applies to the emergency call function, which allows users to choose from preset contacts (e.g., relatives, emergency services, care home). The emergency function can be triggered manually by the user or automatically in the event of a detected fall. The system status also includes an activity dashboard that records movement parameters and user posture during rollator use.

### Ethical aspects

The study was approved by the Ethics Committee of Martin Luther University Halle-Wittenberg (Approval No. 2024-104; letter dated 10 July 2024). Written informed consent was obtained from all participants prior to each focus group session.

## Results

The five focus groups consisted of 15 healthcare professionals (HP) from the fields of nursing, geriatric care, speech therapy, and social pedagogy, as well as 15 rollator users (RU) recruited from day-care facilities. The RU participants were between 67 and 95 years old (mean age 84.3 ± 7.32), with 87% women and 13% men. A core group of six RU participated in both phases of the development process. Based on the participants' feedback, three main categories were identified: (1) use of the rollator in daily life, (2) sensor-based assistance systems, and (3) the application of digital assistive technologies. The anchor quotes for each category are presented in Table 2.

**Table 2:** Overview of categories with anchor examples from rollator users (RU) and healthcare professionals (HP).

Category	Subcategory	Anchor example (translated quotation)	Source
1. Use of a Rollator in Everyday Life	1.1 Hindering conditions	“I can’t manage that anymore. I can’t walk that far”. (A_E_2_2, Pos. 87, T1)	RU
	1.2 Hindering environmental factors	“Yes, all that traffic. And then, as I said, the bad roads here”. (A_E1, Pos. 66–67, T0)	RU
	1.3 Subjective attitudes	“I have my principles, and I’m not changing my daily routines — not even with these modern things I see here”. (A_RH2.Teil, Pos. 60–61, T0)	RU
2. Digital Assistance Systems of a Smart Rollator	2.1 Requirements	“If all that is built in, it just shouldn’t make the rollator any heavier than it already is”. (A_E_2_2, Pos. 223, T1)	RU
	2.2 Sensor-based environmental and posture detection	“Such a function would be useful, yes. Then the accompanying person wouldn’t have to do it”. (A_AH_1, Pos. 124–128, T1)	RU
	2.3 Haptic feedback	“But the vibration — that's important. I like that. The thing with the handles — that's a fine idea”. (A_E_2_1, Pos. 143, T1)	RU
	2.4 User interface	“From my point of view, I’d be so focused on the device that I wouldn’t be looking at the road anymore”. (AE_IG, Pos. 6, T0)	RU
3. Application of Digital Assistance Systems	3.1 Opportunities	“And those who come after us, bit by bit … they’re younger, they’ll get it. When I retire, I’m already good with smartphones — that's a completely different starting point”. (AE_IG, Pos. 21, T0)	HP
	3.2 Barriers	“That's the general problem with digitalisation in Germany — take the electronic patient record, for example. How long it takes to get things like that implemented”. (AB2, Pos. 57, T0)	HP
	3.3 Solutions	“I’d say it's all helpful, but I need to be able to handle it. I need proper training…” (A_AH_2, Pos. 81–90, T1)	RU

### Use of the rollator in daily life

For participants, the rollator represented an essential aid for maintaining or gradually improving personal mobility, both in everyday life and during rehabilitation. Beyond short distances at home or within care facilities, it also supported daily activities such as shopping, walking, and attending medical appointments. However, many users were uncertain about the functional requirements a rollator should meet:

“You really have no idea what to look for the first time you’re confronted with it — at least that's how it was for me.” (A_RH1, Pos. 46, T0)

All participants emphasised the importance of low weight combined with sufficient stability, as well as the inclusion of a storage surface and seat for resting. RU criticised environmental barriers, such as poor ground conditions and heavy traffic, which limited their movement to familiar, perceived-safe routes. They frequently reported that wheels would get caught on uneven surfaces, causing the rollator to tilt and increasing the risk of falls.

From the HP perspective, rollators were considered appropriate aids for regaining mobility and rebuilding muscle strength postoperatively but should also be considered for individuals with gait instability. Proper adjustment and instruction in rollator use prior to application were deemed essential to ensure safe mobility. HP recommended a narrow yet stable design with minimal weight, easy foldability, smooth manoeuvrability, and reliable braking mechanisms to support daily use in both indoor and outdoor settings.

### Sensor-based assistance systems (SAS)

Regarding the SAS features to be developed, participants expressed clear expectations and requirements. All participants agreed that a smart rollator (SR) should include functions such as navigation, environmental detection, and an integrated emergency call system. RU additionally emphasised that integrated lighting, a bell, and an automatic braking function would significantly enhance safety during outdoor mobility. They also requested that the SR remain lightweight yet stable, include shock absorption to reduce shoulder strain, and feature interchangeable wheels adaptable to different terrains. HP highlighted the potential benefits of integrated GPS for user localisation and remote maintenance support in emergencies—particularly valuable for patients with cognitive impairments, who are often prone to disorientation or getting lost. Given the complexity of individual conditions, HP recommended that SAS configurations be easily adjustable and, where possible, voice-controlled. They also suggested coupling auditory feedback with existing hearing aids. Motion monitoring of gait and posture, combined with fall detection and prevention features, was considered particularly useful. Visualising movement data such as walking distance or step count could, according to HP, serve as motivation for physical activity. However, excessive system monitoring could overwhelm or even demotivate users: “If it's constantly monitoring, we’ll drive the poor person standing behind the rollator crazy”. (AB_IG_3). Additional features such as real-time weather alerts were also viewed positively by RU. HP, on the other hand, stressed the importance of built-in safety mechanisms to prevent theft, monitor battery status, and ensure compatibility with other smart systems in the future. Specific requirements were also identified for individual SAS functions. Environmental recognition and posture analysis during rollator use were highly valued and should be complemented by voice feedback in addition to haptic signals when necessary. Classifying objects (e.g., cars, people, e-scooters, bicycles, prams) and environmental conditions (e.g., roads, pavements, grass, kerbs, manhole covers, rails) through a traffic light-style warning system was considered helpful for assessing potential hazards.

From the RU perspective, the navigation function was seen as less relevant for home use or for users with strong spatial orientation and good physical condition. Haptic feedback, however, was rated positively across all groups as a support mechanism for navigation, posture analysis, and hazard warning, as it increased users' awareness and responsiveness: “…What's going on?” (A_RH2, Pos. 234). RU also found warnings for excessive walking speed useful. Most preferred shopping cart–style handles with adjustable positioning. Continuous vibration across the handle was preferred over isolated vibration patterns, provided that each pattern clearly corresponded to a specific function (navigation, warning, etc.). HP cautioned that this distinction was especially important for users with neurological conditions, as excessive stimuli might lead to confusion or cause them to release the rollator. Participants also noted that individuals with sensory impairments might perceive vibrations only weakly—or even find them painful. Therefore, vibration intensity should be individually and unilaterally adjustable. Initially, RU perceived the feedback as too strong, but in later prototype stages, they found it comfortable. Overall, HPanticipated that SAS functionalities would be quickly understood through repeated use and anticipated that future generations of older adults would find handling easier than the current generation.

The user interface (UI) of the app should, according to all participants, be kept simple and avoid excessive information to prevent cognitive overload. To minimise distraction during movement, HP recommended that the display include a “dark mode” and be positioned at eye level so users could keep their gaze on the path ahead rather than looking down. At the start of the project, several RU considered the display size on standard smartphones too small. By contrast, when the first prototype was introduced, the developed UI (see Figure 2) was rated as sufficiently large and clearly visible.

The arrangement of the three core functions—navigation, emergency call, and system status (with integrated movement statistics)—was evaluated positively and found easy to understand. Some RU expressed concern that they might initially focus more on the UI than on their surroundings:

“At the beginning, I’ll probably pay more attention to the app than to the road.” (AE_IG, Pos. 51)

Recording and presenting daily activity data were considered meaningful and motivating by all participants. The integration of a smartwatch extension and physiological parameters (e.g., heart rate, blood pressure) was regarded by HP as particularly useful for emergency situations, such as falls or abnormal vital signs. Integrating the UI into a smartwatch would enable users to operate it independently of the rollator—especially if the rollator had to be left behind due to obstacles. RU noted, however, that adequate visual acuity would be required given the smaller display size.

### Application of digital assistive technologies

With regard to the use of digital assistive technologies such as smart rollators (SRs), three key subcategories emerged from participant feedback: barriers, opportunities, and potential solutions.

#### Barriers

According to participants, one of the main obstacles to adopting digital assistive technologies was the age-related lack of competence and experience in handling digital devices and applications:

“It's not that easy… I’ve never dealt with anything like this before.” (A_AH_2, Pos.139)

Participants also expressed doubts about their ability to learn how to use such technologies:

“I’d have to learn everything from scratch. Will I still be able to understand it? It's all new to me.” (A_AH_2, Pos.137). This was often accompanied by a sense of resistance, fear, and mistrust toward technology and innovative digital tools. Personal safety was considered central to rollator use, with older participants in particular identifying age and physical limitations as possible barriers to using an SR. Some also mentioned a general lack of motivation to engage in physical activity as a deterrent.

Healthcare professionals (HP) raised concerns about potential cognitive overload among users, given the volume of information processed by the various SAS components, especially in view of reduced reaction times and conditions such as dementia or post-stroke impairments.

Beyond user-related challenges, participants highlighted broader systemic barriers, including regulatory and administrative constraints in Germany [e.g., Medical Devices Implementation Act (MPDG)] and delays in national digitalisation efforts:

“That's the general problem in Germany with digitalisation—take the electronic patient record, for example. How long it takes to get things like that implemented.” (AB2, Pos. 57)

The cost of a smart rollator was also cited as a major limiting factor for widespread adoption.

#### Solutions and facilitating factors

Despite these challenges, participants identified several possible solutions for improving acceptance and usability. All participants agreed that a simple and intuitive user interface (UI) and straightforward SAS functions, which could be individually activated or deactivated as needed, were essential. An integrated voice control function was considered valuable both as an interaction method and as an emergency aid.

The modular design of the SAS was viewed positively, as it prevents excessive increases in the rollator's overall weight and avoids structural modifications that might conflict with regulatory requirements. The modular system also enables easy removal of components, thereby reducing theft risk. All participants emphasised the importance of initial training or orientation sessions for both users and their relatives, ideally provided by suppliers or medical supply stores. Additionally, the UI should include short tutorials explaining SAS functions and settings, allowing users to solve minor problems independently. From the RU perspective, practical training sessions or repeated practice before first use were recommended to familiarise users with the system and its functions.

A gamification feature was implemented in the UI during the development process, displaying daily activity statistics to motivate users toward greater movement: “Oh, that's something—a challenge!” (A_AH_2, Pos. 88–90, T1).

#### Opportunities

HP saw the main opportunities of SR use in reducing workload across nursing, therapy, and inpatient care settings: “Those who need to go for an x-ray or a CT or ECG and can’t find the way—this is great. It's an offer. You save staff”. (AB2, Pos. 46–48, T0). HP also pointed out that SRs equipped with appropriate SAS could offer added value in diagnostics and therapy. Furthermore, integrating other smart technologies—such as smartwatches—or connecting SRs to existing smart home environments was seen as promising. All participants agreed that SRs could enhance safety, mobility, and autonomy for older adults, representing an important step toward supporting independence and well-being in later life.

## Discussion

The results of this study demonstrate that digital and sensor-based extensions of conventional rollators are generally perceived positively by older users, provided that they enhance perceived safety and independence without compromising ease of use. These findings are consistent with those of Werner et al. (52), who showed in their study on sit-to-stand assistance among older adults that technological support systems are accepted when experienced as intuitive, stable, and trustworthy. A key determinant of acceptance—both in their study and in the present one—was the simplicity and comprehensibility of operation, along with the subjective sense of control over the technology. This aspect was repeatedly requested by both rollator users and nursing professionals at an early stage of the development process, particularly with regard to the design of haptic feedback and the user interface, taking into account individual health impairments or illnesses. Furthermore, the feedback from rollator users and nursing professionals aligns with the findings of Dupret et al. (31), who evaluated the *DigiRoll* and reported positive perceptions of its safety features (lighting, fall detection, emergency call system). All Participants described a marked increase in their sense of safety, coupled with a desire for individualisation and adaptability depending on their own needs and state of health. Taking health status and the resulting individual needs into account reflects the demands made by Mostofa et al. (25) and Martins et al. (37) when it comes to the development of assistance systems. Dupret et al. also emphasised that new assistive functions may have symbolic effects, such as “modernising” the aid and reducing the stigma associated with rollator use. Such psychosocial effects are also reflected in the present findings, where participants reported increased motivation and a renewed sense of autonomy.

In this study, RU primarily highlighted design limitations in existing rollator models and identified opportunities for ergonomic optimisation to improve everyday usability and handling.At the same time, many RU expressed openness towards digital assistance functions. This apparent ambivalence may reflect age-related differences in digital experience, whereby limited familiarity with technology coexists with a willingness to engage with supportive innovations.

Comparable findings were reported by Orenius et al. (39), who observed that experienced RU draw on practical knowledge when interacting with smart rollators (SR), and that a subgroup demonstrates particular interest in sensor-based assistance systems (SAS) and readiness to acquire new skills. Similarly, Aruona et al. (42) found that RU criticised the physical strain associated with inadequately designed rollators and questioned their suitability for outdoor use without technical enhancements.

In contrast, healthcare professionals (HP) more frequently articulated system-oriented concepts, including navigation systems, GPS tracking, voice control, and integration with other digital devices. Beyond enhancing user support, these proposals also reflected considerations regarding care coordination and organisational efficiency. The assistance functions suggested in the present study align with recommendations reported by Aruona et al. (42) and correspond to technical implementation approaches such as those described by Bieber et al. (24).

A more differentiated analysis of the perspectives of RU and HP reveals both convergences and divergences. While both groups emphasised safety, ease of use, and individual adaptability as central requirements, RU focused more strongly on lived-experience aspects such as ergonomics and daily handling. In contrast, HP placed greater emphasis on system integration and implications for care delivery. The shared prioritisation of safety, however, underscores a common understanding of assistive technologies as tools for promoting independence and security in older age. Werner et al. (38) had already pointed out the methodological and conceptual shortcomings of earlier studies on smart rollators—particularly the lack of standardisation, small sample sizes, and limited user involvement. The participatory approach adopted in the present study directly addresses these gaps by integrating user needs and perspectives in the early stages of development. Hofstetter et al. (53) likewise emphasised that participatory design processes are crucial for preventing acceptance barriers and enhancing the clinical relevance of digital assistance systems. Nursing professionals play a key role as mediators between technical development and practical application—a role ensured in this study through their active participation in the focus groups.

In terms of technical implementation, the present findings parallel those of Wolf-Ostermann und Rothgang (54), who underscored the need for systematic integration of digital innovations into healthcare structures. Technological acceptance alone is insufficient; successful implementation requires training, technical support, and clear responsibility within the care system. These insights are consistent with the participants' (RU & HP) suggestions for structured training programmes, technical guidance, and modular retrofit options.

Furthermore, data from the German National Association of Statutory Health Insurance Funds (GKV) (55) highlight the societal relevance of digital care innovations. The GKV stresses that assistive technologies can meaningfully relieve the care sector only when introduced in a socially equitable, practice-oriented, and educationally supported manner. The present study reinforces this perspective by showing that both the involvement of end users and training opportunities for professionals and relatives are essential prerequisites for acceptance and sustainable use. In line with the strategic goals outlined in the GKV series, the approach presented here exemplifies practice-oriented, interdisciplinary innovation in the care sector.

Overall, the findings indicate that smart rollators hold substantial potential for promoting mobility, safety, and quality of life in older age—provided they are developed in a user-centred manner, tailored to individual needs, and embedded within existing care processes. The integration of sensor-based assistance systems (SAS) for real-time movement monitoring also offers diagnostic potential for physicians, therapists, and nursing staff to assess users' health status and derive therapeutic recommendations, as also suggested by Fernandez-Carmona et al. (43). Participants viewed the potential integration of SRs into smart ecosystems and their coupling with other smart technologies (e.g., wearables) positively, although technical implementation was not feasible within the current project. Incorporating wearable-based physiological parameters could substantially enhance health monitoring and, combined with the SR's emergency call function, provide critical support in emergencies. Thus, the SR has the potential to serve not only as an assistive aid but also as an interventional device that promotes and maintains physical activity in both rehabilitation and everyday contexts (37) thereby alleviating the workload of healthcare professionals (37). Morone et al. (35) demonstrated that gait training with a smart rollator in patients with subacute stroke led to greater improvements in balance, walking distance, and gait quality than training with conventional rollators. Future studies should therefore evaluate the developed SAS under real-world conditions, targeting specific user groups, and subsequently investigate additional patient populations within clinical and rehabilitative contexts that may benefit from such systems.

The approach proposed by Sierra et al. (34)—enabling social interaction between users and SRs via an appropriate user interface—represents an ideal complement to the purely technical-assistive functions, as it addresses the psychosocial well-being of the user. This aligns with our findings, where RU considered a voice-interaction function particularly useful and expressed a preference for companionship during daily activities.

Future work should also assess, following proof of validity, the extent to which these cost-effective modular SAS can be implemented within existing legal frameworks and whether financial support or reimbursement from health insurance providers is feasible. For assistive technologies to meaningfully relieve the care sector and improve quality of life for older adults, it is essential to raise awareness among professionals, institutions, caregivers, and affected individuals regarding their availability and benefits (3).

## Limitations

The methodological design of this study followed a design-based research (DBR) approach characterised by iterative development and reflection cycles. This process made it possible not only to explore user needs but also to feed them back into technical development over multiple iterations. The chosen approach thus responds directly to the calls of Werner et al. (38) for greater user-centeredness and methodological consistency.

By combining empirical data collection and design processes, the study achieved high ecological validity, as research and development were closely intertwined in real-world contexts. The combination of focus groups involving both rollator users and nursing professionals enabled a multi-perspective analysis of needs, acceptance factors, and potential barriers. This approach corresponds with the recommendations of Hofstetter et al. (53), who emphasised that participatory dialogue between end users and professionals is crucial for the usability and practical feasibility of digital aids. Similar to Dupret et al. (31) the present study confirms that qualitative methods are particularly well suited to capturing subjective meanings, emotions, and motivational aspects that are central to acceptance decisions.

Nevertheless, the qualitative design entails certain methodological limitations. The sample size was limited to 30 participants, all recruited from specific care facilities, which restricts generalisability and representativeness. Despite multiple reflection and validation procedures social desirability bias in group discussions cannot be entirely excluded. Given the qualitative and context-specific nature of the design-based research approach, the findings cannot be generalised to all rollator users or healthcare settings. Rather, they should be understood as exploratory and hypothesis-generating. Future research should therefore include larger and more diverse samples and incorporate quantitative and longitudinal designs to empirically validate usability, effectiveness, and clinical impact under real-world conditions. In order to minimise the risk of social desirability bias, all participants were informed that their statements would be anonymised during transcription and analysis, and that critical comments were explicitly welcometo identify potential weaknesses in the prototype development. The aim was to foster an open and trusting atmosphere by conducting the focus groups in familiar settings and ensuring neutral moderation of the discussions, using a carefully designed, non-leading interview guideand applying indirect questioning techniques where appropriate to facilitate candid responses on sensitives issues.In conclusion, the participatory and iterative research design of this study provides a robust methodological foundation for the development of smart rollators. The combination of qualitative depth analysis, practice-based testing, and direct feedback into technical development represents a valuable model for future research and innovation projects. Future studies should build on these findings using longitudinal, interdisciplinary designs to evaluate the long-term effectiveness and practical implementation of smart mobility aids in everyday care contexts.Future research should examine additional use cases (e.g., hospital settings) and broader user groups with varying health conditions, ideally based on larger and more diverse samples. To more robustly assess the effectiveness of such sensor-based assistance systems (SAS), end users should engage with the technology over extended periods. Particular emphasis should be placed on evaluating system performance under real-world conditions, including diverse terrains and environmental contexts.

Furthermore, the analysis of system-generated data (e.g., mobility, vital, or postural parameters) should be systematically explored with regard to its potential for preventive and rehabilitative applications. Finally, future studies should investigate the transferability of SAS components to other mobility aids, such as wheelchairs or walking sticks, and evaluate which user populations may benefit most from such adaptations.

## Data Availability

The original contributions presented in the study are included in the article/Supplementary Material, further inquiries can be directed to the corresponding author.
